# Changes in mortality patterns and place of death during the COVID-19 pandemic: A descriptive analysis of mortality data across four nations

**DOI:** 10.1177/02692163211040981

**Published:** 2021-08-23

**Authors:** Sean B O’Donnell, Anna E Bone, Anne M Finucane, Jenny McAleese, Irene J Higginson, Stephen Barclay, Katherine E Sleeman, Fliss EM Murtagh

**Affiliations:** 1 Wolfson Palliative Care Research Centre, Hull York Medical School, University of Hull, Hull, UK; 2 Cicely Saunders Institute of Palliative Care, Policy & Rehabilitation, King’s College London, London, UK; 3 Clinical Psychology, University of Edinburgh, Edinburgh, UK; 4 Patient and Public Involvement Partner, York Hospitals NHS Foundation Trust, Harrogate, UK; 5 Department of Public Health and Primary Care, University of Cambridge, Cambridge, UK

**Keywords:** Palliative care, terminal care, COVID-19, pandemics, mortality, residential facilities, place of death

## Abstract

**Background:**

Understanding patterns of mortality and place of death during the COVID-19 pandemic is important to help provide appropriate services and resources.

**Aims:**

To analyse patterns of mortality including place of death in the United Kingdom (UK) (England, Wales, Scotland and Northern Ireland) during the COVID-19 pandemic to date.

**Design:**

Descriptive analysis of UK mortality data between March 2020 and March 2021. Weekly number of deaths was described by place of death, using the following definitions: (1) expected deaths: average expected deaths estimated using historical data (2015–19); (2) COVID-19 deaths: where COVID-19 is mentioned on the death certificate; (3) additional non-COVID-19 deaths: above expected but not attributed to COVID-19; (4) baseline deaths: up to and including expected deaths but excluding COVID-19 deaths.

**Results:**

During the analysis period, 798,643 deaths were registered in the UK, of which 147,282 were COVID-19 deaths and 17,672 were additional non-COVID-19 deaths. While numbers of people who died in care homes and hospitals increased above expected only during the pandemic waves, the numbers of people who died at home remained above expected both during and between the pandemic waves, with an overall increase of 41%.

**Conclusions:**

Where people died changed during the COVID-19 pandemic, with an increase in deaths at home during and between pandemic waves. This has implications for planning and organisation of palliative care and community services. The extent to which these changes will persist longer term remains unclear. Further research could investigate whether this is reflected in other countries with high COVID-19 mortality.

**What is already known about the topic?**

- The COVID-19 pandemic has led to excess mortality globally.
- Mortality has been high in the UK, compared to other nations. However, place of death – and particularly any changes in deaths at home during and between COVID-19 waves – has not yet been reported in detail.

***What this paper adds***

- Number of people who died at home in England, Wales, Scotland and Northern Ireland have remained constantly above expected levels throughout the first 12 months of the COVID-19 pandemic, with an increase of 67% in the first wave, 43% in the second wave and 33% between these waves.
- People who died in care homes increased above expected levels in England, Wales, Scotland and Northern Ireland by 134% during the first wave and 10% in the second wave of the pandemic but fell below expected levels by 3% between these waves.
- People who died in hospitals in England, Wales, Scotland and Northern Ireland increased by 35% above expected levels in the first wave of the pandemic, and 26% in the second wave, but fell by 13% between these waves.

***Implications for practice, theory or policy***

- Further research is needed to understand the reasons for this increase in numbers of people who died at home, whether this will be sustained in the future, and if this has been replicated in other countries.
- There are implications for future organisation of services and resource allocation. If increase numbers of home deaths continue, increased resources are needed to support primary, community and palliative care services.
- It is important for primary care, community and palliative care services to be well-integrated and efficient in providing support in patient’s homes and care homes.

## Background

COVID-19 has led to high excess mortality globally, with healthcare systems and economies in both developed and developing countries placed under significant pressure. The key role of palliative and end-of-life care has been emphasised. However, providing high quality palliative care has been difficult due to the challenges posed by the pandemic^1,2^ and services offering palliative care report feeling ignored during the pandemic response.^3^ COVID-19 has a rapid course^4^ and requires rapidly responsive services. The experience of those involved in caring for patients at the end-of-life during the pandemic, irrespective of their illness, has been that communication, support and advance care planning have been vitally important, with patients and loved ones often highly distressed during a time of social isolation and visiting restrictions.^5,6^

To learn from this crisis, governments, policymakers and healthcare providers need to know how the demographics of mortality, including place of death, have changed. Given the shift in mortality patterns brought about by the pandemic, further study of numbers and patterns in place of death is required to highlight implications for palliative and end-of-life care provision in the UK. A study by Bone et al.^7^ conducted during the first 10 weeks of the pandemic in England and Wales demonstrated a 220% increase in care home deaths, while home and hospital deaths increased by 77% and 90% respectively.

The aim of this study is to analyse the patterns of mortality, with an emphasis on place of death, in the United Kingdom (UK) during the initial 12 months of the COVID-19 pandemic to suggest implications for palliative and end-of-life care service provision, resource allocation and further research.

## Methods

### Design

Descriptive analysis of mortality data routinely collected in England, Wales, Scotland and Northern Ireland. The methods outlined by Bone et al.^7^ were used in the design of this study.

### Data sources

Publicly available datasets for the study timeframe (7/3/2021–12/3/2021 in England, Wales and Northern Ireland, and 9/3/2021–14/3/2021 in Scotland) published online by the Office for National Statistics (ONS), National Records of Scotland (NRS) and Northern Ireland Statistics and Research Agency (NISRA) were used as follows:

- Estimates of the population for the UK, England and Wales, Scotland and Northern Ireland^8^
- Weekly registered deaths in England, Wales, Scotland and Northern Ireland including COVID-19 deaths and historical weekly deaths^9–12^
- Five-year average deaths registered by place of death for England, Wales, Scotland and Northern Ireland^13–16^

Data were described by week, where week 1 is the first week of the year. Death registration data differed between nations; see Supplemental Appendix Text 1 for details.

### Analysis

The following mortality categories were calculated from the datasets:

1. *Expected deaths*: Deaths expected to occur in a typical year without the pandemic. This was derived from the average number of deaths over the previous 5 years (2015–2019) obtained from the national statistics agencies and used to calculate average deaths separated into five age groups (<45, 45–64, 65–74, 75–84 and 85+ years).
2. *COVID-19 deaths*: Deaths where COVID-19 was mentioned on the death certificate as published in the datasets. COVID-19 can be mentioned on the death certificate based on clinical presentation in the absence of a positive test.^17^
3. *Additional non-COVID-19 deaths*: Deaths in excess of expected deaths, not accounted for by deaths where COVID-19 is mentioned on the death certificate.
4. *Baseline deaths*: The total number of deaths registered in each week minus the number of COVID-19 and additional non-COVID-19 deaths.

To compare patterns between the UK nations and regions, death rates for each mortality category above were calculated per 100,000 of population, using population estimates from mid-2019.^8^

Due to a lack of consensus on a definition to describe the waves of the pandemic, we defined a wave as:

- 10% or more increase in total deaths, compared to expected deaths, sustained for at least 3 weeks or more and ending when total deaths were <10% above 5-year average for at least 3 weeks, where the first week of this 3-week period is the wave end date.

### Place of death

Place of death was described using the definitions provided in the datasets of each nation (Supplemental Appendix Table 1).

Weekly data for 5-year average deaths by place of death was used to calculate baseline and additional deaths in each location and in the four nations combined.

### Patient public involvement (PPI)

Our PPI partner (JMcA), a co-author of this work, was active in discussions of the data and its presentation, including the decisions regarding key messages of the work. JMcA was also involved in revisions of the drafts and approval of the final manuscript.

### Ethics and consent

This analysis was conducted using publicly available, anonymised datasets. Therefore, no ethical approval was necessary.

## Results

**Figure 1.**
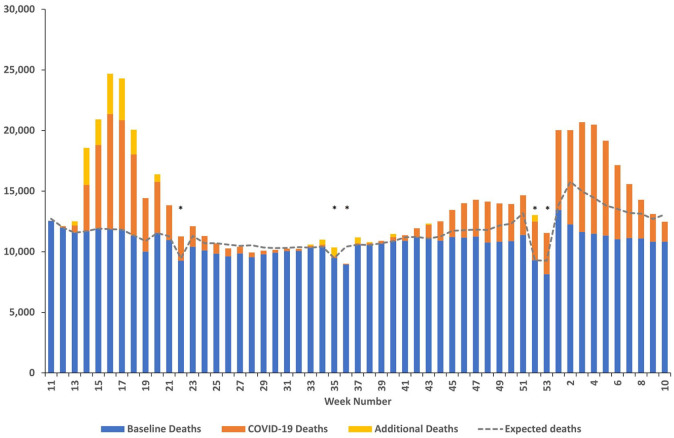
All deaths registered in the UK during the COVID-19 pandemic between week 11 of 2020 and week 10 of 2021 (7/3/2020–12/3/2021 in England, Wales and Northern Ireland, and 9/3/2020–14/3/2021 in Scotland). *Bank and public holidays in 2020/21, and historically, may have impacted death registrations during the indicated weeks.

In the UK between week 11 of 2020 and week 10 of 2021 (7/3/2020–12/3/21 in England, Wales and Northern Ireland, and 9/3/2020–14/3/2021 in Scotland), there were 798,643 deaths registered, an increase of 119,241 (17.6%) compared to expected deaths. 147,282 (18.4%) of these deaths had COVID-19 mentioned on the death certificate and there were 17,672 additional non-COVID-19 deaths (Figure 1). There were differences in patterns of mortality between the UK nations and regions of England over the study period (Supplemental Appendix Table 2, Supplemental Appendix Figures 1 and 2, Supplemental Appendix Text 2).

Using our definition, two waves of deaths are evident:

- Wave 1 = weeks 14–23 of 2020 (28/3/2020–30/5/2020 in England, Wales and Northern Ireland 30/3/2020–1/6/2020 in Scotland)
- Period between wave 1 and wave 2 = weeks 23–43 of 2020 (30/5/2020–17/10/2020 in England, Wales and Northern Ireland 1/6/2020–19/10/2020 in Scotland)
- Wave 2 = week 43 of 2020–week 8 of 2021 (17/10/2020–20/2/2021 in England, Wales and Northern Ireland, and 19/10/2020–22/2/2021 in Scotland)

### Mortality patterns by age

**Figure 2.**
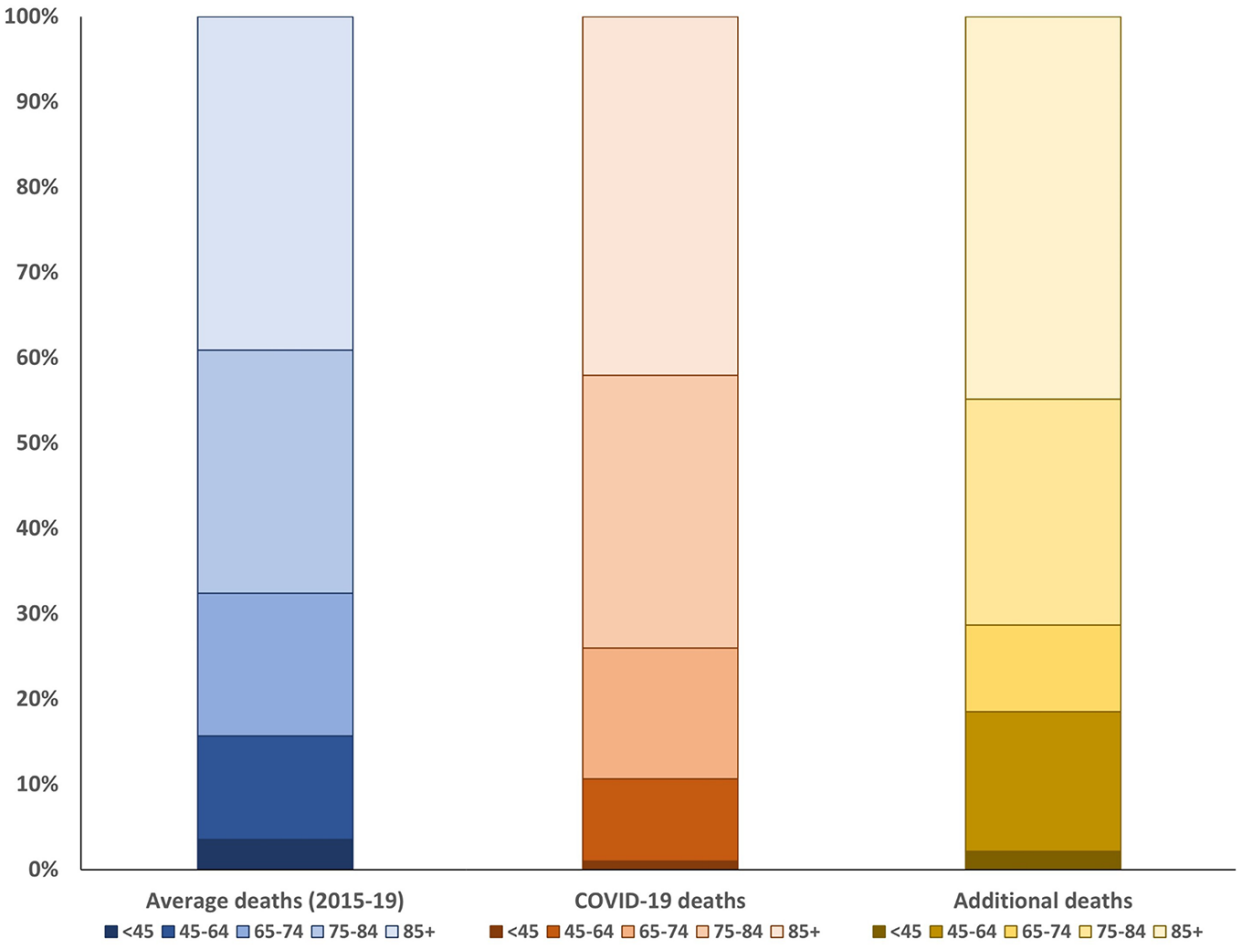
Age distributions of baseline deaths, COVID-19 deaths and additional non-COVID-19 deaths between week 11 of 2020 and week 10 of 2021 (7/3/2020–12/3/2021 in England, Wales and Northern Ireland, and 9/3/2020–14/3/2021 in Scotland).

The age distributions of people who died in the UK showed COVID-19 deaths were overall older compared to expected deaths (Figure 2 and Supplemental Appendix Table 3). The age distribution of COVID-19 deaths was similar between waves one and two, and between these waves (Supplemental Appendix Table 3). Data on the age distribution of people who died in England and Wales, Scotland and Northern Ireland is shown in Supplemental Appendix Table 3.

### Place of death during the pandemic

**Figure 3.**
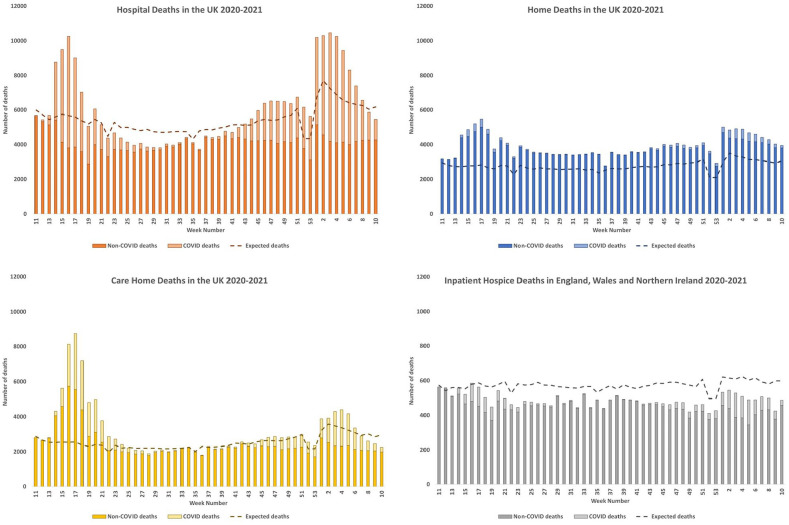
COVID-19 and additional non-COVID-19 deaths registered as occurring in hospitals, homes and care homes in England, Wales, Scotland and Northern Ireland, and inpatient hospices in England, Wales and Northern Ireland during the COVID-19 pandemic between week 11 of 2020 and week 10 of 2021 (7/3/2020–12/3/2021 in England, Wales and Northern Ireland, and 9/3/2020–14/3/2021 in Scotland) according to place of death and geographical area.

Where people died changed during the pandemic in England, Wales, Scotland and Northern Ireland compared to the expected place of death (Figure 3 and Table 1). Overall during the study timeframe, compared to expected deaths, the number of people who died at home increased by 41%, in care homes increased by 23% and in hospital increased by 11%. Fewer people died in inpatient hospices in England, Wales and Northern Ireland amounting to a reduction of 15% overall (Table 1). A summary of the changes in place of death by nation is presented in Supplemental Appendix Figures 3–7 and Supplemental Appendix Text 3.

**Table 1.** Percentage of deaths occurring in homes, hospitals and care homes in England, Wales, Scotland and Northern Ireland, and in inpatient hospices in England, Wales and Northern Ireland overall, during the two waves and between these waves of the COVID-19 pandemic.

Place of death	Wave 1^a^			Between Wave 1 and Wave 2^b^			Wave 2^c^			Analysis period		
	% Of total deaths	% Of COVID-19 deaths	% Change in deaths versus 5-year average	% Of total deaths	% Of COVID-19 deaths	% Change in deaths versus 5-year average	% Of total deaths	% Of COVID-19 deaths	% Change in deaths versus 5-year average	% Of total deaths	% Of COVID-19 deaths	% Change in deaths versus 5-year average
Home	25	5	+67	33	6	+33	27	6	+43	29	6	+41
Hospital^d^	40	62	+35	40	63	−13	48	72	+26	45	68	+11
Care home^d^	31	31	+134	20	29	−3	20	20	+10	23	24	+23
Inpatient hospice^e^	3	1	−8	4	2	−16	3	1	−18	4	1	−15
Other^d^	2	1	+55	3	1	+20	2	1	+35	2	1	+31
All^f^			+62			+1			+21			+16

Percentage change in deaths compared to the 5-year average (2015–2019) over the same period in these locations. Between week 11 of 2020 and week 8 of 2021 (7/3/2020–20/2/2021 in England, Wales and Northern Ireland, and 9/3/2020–22/2/2021 in Scotland).

a Wave 1 = week 14–23 of 2020 (28/3/2020–30/5/2020 in England, Wales and Northern Ireland, 30/3/2020–1/6/2020 in Scotland) – 9 weeks total length.

b Between wave 1 and wave 2 = week 23–43 of 2020 (30/5/2020–17/10/2020 in England, Wales and Northern Ireland, 1/6/2020–19/10/2020 in Scotland) – 20 weeks total length.

c Wave 2 = week 43 of 2020–week 8 of 2021 (17/10/2020–20/2/2021 in England, Wales and Northern Ireland, 19/10/20–22/2/2021 in Scotland) – 18 weeks total length.

d Data for inpatient hospice deaths in Scotland may be registered in these location categories.

e Data available for England, Wales and Northern Ireland only.

f Data for England and Wales does not include deaths of non-residents therefore this total underestimates total UK deaths.

When comparing the two waves of the pandemic (Figure 3 and Table 1), where people died in the four UK nations varied. Compared to expected deaths, those who died in care homes increased by 134% in the first wave and 10% in the second wave. There was a 35% increase in deaths registered in hospitals in the first wave, and a 26% increase in the second wave. Between wave 1 and wave 2, deaths in care homes fell by 3% and deaths in hospitals by 13%.

In England, Wales, Scotland and Northern Ireland, there was a persistent increase in people who died at home compared to expected, throughout both waves and between these waves (Figure 3 and Table 1). In the first wave, deaths at home increased by 67% and this increase was maintained between the waves by 33%, and in the second wave by 43% above expected.

Deaths where COVID-19 was reported on the death certificate contributed to the increase in hospital and care home deaths to a much greater extent than home deaths. The percentage of deaths during the study period occurring in hospitals and care homes in the UK where this was the case was 32% and 21% respectively. Of those who died at home, just 4% had COVID-19 mentioned on the death certificate. Therefore, most of the excess deaths occurring at home during the study period were non-COVID-19 deaths as can be seen in Figure 3.

**Figure 4.**
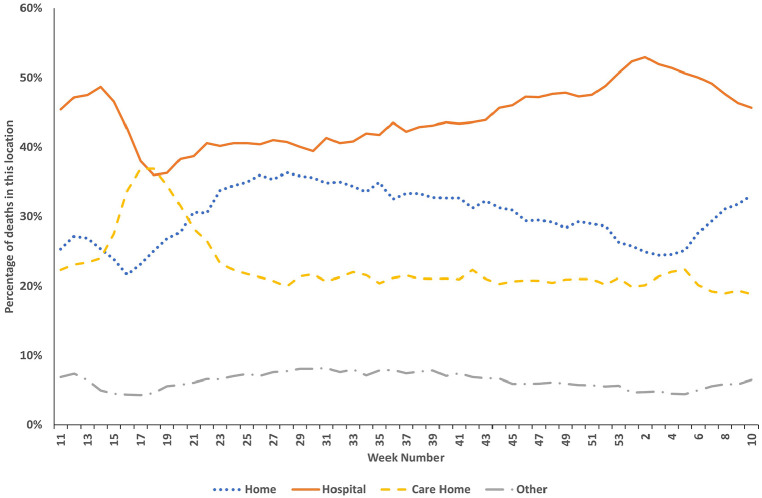
All deaths registered in the UK (England, Wales, Scotland and Northern Ireland) during the COVID-19 pandemic between week 11 of 2020 and week 10 of 2021 (7/3/2020–12/3/2021 in England, Wales and Northern Ireland, and 9/3/2020–14/3/2021 in Scotland) according to place of death by relative proportion.

There were also variations in the relative proportions of place of death throughout the pandemic (Figure 4 and Table 1). Over the study period in the UK, the percentage of all registered deaths occurring in hospital was 45%, at home was 29% and in care homes was 23%. In England, Wales and Northern Ireland, those who died in inpatient hospices accounted for 4% of all deaths during the study timeframe. Care homes became the most common place to die in the UK in week 18 during the first wave of the pandemic before returning to be the third most common place to die by week 21. There are differences in the relative proportions of place of death between the nations of the UK; these are shown in Supplemental Appendix Figure 7 and summarised in Supplemental Appendix Text 3.

## Discussion

Our analysis of UK mortality data has shown that, overall, there has been an 17.6% increase in deaths during the first 12 months of the pandemic. We have been able to define two distinct pandemic waves: from March 2020 to the end of May 2020, and from mid-October 2020 to the end of February 2021. We already know that the numbers of people dying in hospitals, and care homes increased during these pandemic waves. However, we newly report the sustained increase in people who died at home not just during these waves, but between these waves.

Deaths at home were persistently elevated throughout the whole 12 months of the pandemic, while hospital and care home deaths do not show this pattern. This highlights the need for reorientation of palliative and end-of-life care towards the community. Previous research has shown that most people who state a preference would prefer to die at home, although many do not state any preference.^18,19^ However, the pandemic may have impacted on patient and family/carer decision-making regarding preferred place of care and therefore our previous understanding of this requires updating.

During the first wave of the pandemic, those who died in hospitals, care homes and at home all increased, with the greatest relative increase being in care homes. Consequently, care homes became the most common place for people to die for a brief period during the first wave. Due the need to shield residents from COVID-19, and resource limitations such as a lack of adequate personal protective equipment, palliative and end-of-life care provision for those occupying the 539,900 care home beds in the UK^20–23^ (Supplemental Appendix Table 5) has been challenging.^24^ Despite a decrease in care home deaths in the second wave, care homes remain an important place of care for people at the end-of-life and it is essential to ensure they are adequately resourced and integrated into the wider palliative care system.

The rise in people who died at home, and the need for greater care home support, has increased demand on community services and prompted shifts in how care is delivered. The changes brought about due to the pandemic such as the increased use of advance care planning^25^ supported by the development of resources to aid such conversations,^26,27^ and the use of virtual technologies to support patients and family members^28^ may well continue in future practice. In addition, visiting restrictions and social distancing has emphasised the importance to patients of face-to-face visits of both family members and healthcare workers, especially at the end-of-life.^29^

The reasons for persistently elevated deaths at home between the first and second pandemic waves is unclear and the majority of those who died at home did not have COVID-19 mentioned on the death certificate; this indicates that factors other than infection with SARS-CoV-2 may have contributed to this rise. For example, interruption in care or missed opportunities for intervention, for example in those with acute myocardial infarction or symptoms suggestive of cancer,^30–32^ may have increased the number of people dying at home from non-COVID-19 causes. This may contribute to an ongoing increase in mortality as the incidence of COVID-19 falls. However, deaths occurring outside of hospital may have been under recognised as caused by COVID-19 with suggestion that reporting of less well-defined causes of death on the death certificate may represent underreported COVID-19.^33^

Prior to the pandemic, hospitals were often seen as the safer place of care due to the availability of rapid medical support^34^ however, the fear of nosocomial infection as well as visiting restrictions and capacity issues may have increased reluctance to accept inpatient admission. This reduction in choice of place of care may have influenced patient and family member preferences.^35^ Even before the pandemic, evidence suggested that increasing numbers of people are expected to die at home in the next decade in Britain.^36,37^ Therefore, primary care, palliative care and other services may see increased demand for end-of-life care at home with the acknowledgement that the COVID-19 pandemic is a historically unique and hopefully temporary anomaly and there is uncertainty regarding long term impacts. Further research is needed to explore whether societal preferences and expectations have changed, how these trends will continue, and the implications this will have for services.

A recently published analysis by Wu et al.^33^ also analysed official death registrations to the end of June 2020 in England and Wales. This analysis incorporated data on place of death but also provided information on cause of death allowing for greater evaluation of the possible explanations for additional deaths in the locations studied. Our analysis builds on this data by extending the study period to include the entirety of the two waves of the pandemic and the inter-wave period across all four nations of the UK. However, further analysis including the data used by Wu et al. exploring cause of death across this period in the locations outlined would be helpful to further evaluate the possible reasons for persistently elevated non-COVID deaths at home.

Throughout the pandemic, people who died in inpatient hospices fell to below expected levels; there appears to have been decreased demand on inpatient palliative care units with a simultaneous reduction in capacity because of COVID-19 restrictions. The rise in hospital and community deaths indicates that need for palliative care services in these locations have increased. Studies have highlighted marked changes in palliative care services during the pandemic.^3,28,38,39^ It will be important to address the barriers highlighted by these studies, including remote consultations, service adaptations and increased community resources.

Additional non-COVID-19 deaths were higher during the first wave, but not in the second wave. As the pandemic progressed, testing capacity increased (Supplemental Appendix Figure 7), as did understanding of the condition, meaning it is likely cases of COVID-19 were less often recognised earlier in the pandemic. Particularly in the first wave, the reduced availability of testing, with resources focussed on hospital inpatients, may have resulted in an underreporting of COVID-19 in the community.

### Strengths and limitations

This study has described the patterns of mortality and place of death during the COVID-19 pandemic using official death registration data for whole country populations. There are however limitations to the study arising from the data sources and the methods used.

There were differences in the reporting of the data and the definitions used between the three national statistics agencies, including variation in week numbering, place of death definitions and variation over whether deaths of those resident outside the country or region of death were included in the data (Supplemental Appendix Text 1). ‘Hospice’ (referring to inpatient hospices) was a separate location category in England, Wales and Northern Ireland but has been combined with other categories in Scotland. Given the relatively small numbers of inpatient hospice deaths, this is likely to have had minimal impact on the overall UK data patterns described. In addition, delays in reporting of deaths such as during bank holidays appears to have impacted our data during those weeks and have also impacted the historical death data however, this occurs in isolated weeks and is unlikely to have a significant impact on the overall data presented. There may also be secular trends occurring between years in the historical data used.

## Implications for policy, data and future research

There has been a shift in where people have died during the pandemic, with a persistent and sustained increase in people who died at home throughout the pandemic period. Further research is required to understand the reasons for this. Understanding the decision-making process around place of care from the perspective of family and/or carers of those who died during the pandemic is especially important; to understand why this has happened and whether the changes are likely to continue in the future. To gain an understanding of these patterns, further research employing data on an individualised rather than aggregated whole population level will be required.

Studies in other areas of the world with high levels of excess mortality are also needed to assess whether this pattern is replicated elsewhere. Further analysis of additional non-COVID-19 deaths by disease group would enable assessment of the true number and nature of these deaths. The importance of greater consistency in mortality data collection and reporting between nations has also been highlighted.

Our paper highlights place of death for those dying with COVID-19, and also how the pandemic has changed place of death for those dying from non-COVID-19 causes. International comparisons of place of death are challenging^40^ and evidence suggests significant variations in place of death between countries.^41^ However, within-country analyses of changes in place of death over time and during the pandemic are important.

## Conclusions

Where people died in England, Wales, Scotland and Northern Ireland changed during the COVID-19 pandemic, affecting not only those with COVID-19 but also people who died from other causes. There has been a sustained rise in people who died at home, not just during the pandemic waves but between these waves as well. Community, primary and palliative care services need to be adequately resourced to meet current and future palliative care needs, and to be flexible and responsive if further changes in place of care and death occur. Further research is needed to understand the reasons for these changes in place of death, whether they will be sustained in the future and if they are replicated elsewhere in areas with high levels of excess mortality.

## Supplemental Material

sj-pdf-1-pmj-10.1177_02692163211040981 – Supplemental material for Changes in mortality patterns and place of death during the COVID-19 pandemic: A descriptive analysis of mortality data across four nations Supplemental material, sj-pdf-1-pmj-10.1177_02692163211040981 for Changes in mortality patterns and place of death during the COVID-19 pandemic: A descriptive analysis of mortality data across four nations by Sean B O’Donnell, Anna E Bone, Anne M Finucane, Jenny McAleese, Irene J Higginson, Stephen Barclay, Katherine E Sleeman and Fliss EM Murtagh in Palliative Medicine
