# Supplementary material for: Changes in mortality patterns and place of death during the COVID-19 pandemic: A descriptive analysis of mortality data across four nations
Source: Palliat Med. 2021 Aug 23;35(10):1975–84. doi: 10.1177/02692163211040981 (PMC8641034; doi:10.1177/02692163211040981)
Supplement: sj-pdf-1-pmj-10.1177_02692163211040981 – Supplemental material for Changes in mortality patterns and place of death during the COVID-19 pandemic: A descriptive analysis of mortality data across four nations [file sj-pdf-1-pmj-10.1177_02692163211040981.pdf]

Supplemental Material

Appendix Text 1. Outline of the alterations in numbering of weeks used in the data for the analysis

The datasets were numbered according to week number; the ONS data for England and Wales and the NIRS data for NI was numbered according to weeks ending on a Friday. The ONS dataset started with week 1 ending 03/01/2020 and NIRS started with week 1 ending 10/01/2020. Therefore, the NIRS dataset reported weeks numbering one behind that of the ONS data. The NIRS data was therefore renumbered to conform to the week numbering convention of the ONS data, starting week 2 ending 10/01/2020. The NRS data for Scotland was numbered according to weeks ending on a Sunday, starting from week 1 ending 30/12/2020. This means that, compared to the ONS and NIRS data, the data for a given week excludes two days from the previous week and includes two days from the subsequent week.

The data for England and Wales includes registered deaths of those resident in those nations only. Whereas the data for Scotland and Northern Ireland reports all deaths registered in those nations irrespective of usual residence.

Appendix Table 1. Definitions used by the national statistics agencies to code the location of deaths

England and Wales[1]	Scotland[2]	Northern Ireland[3]
Hospital (acute or community, not psychiatric)	Hospital (NHS hospitals)*	Hospital
Deaths at home are those at the usual residence of the deceased (according to the informant), where this is not a communal establishment.	Home/Non-institution	Home
Care homes includes homes for the chronic sick; nursing homes; homes for people with mental health problems and non-NHS multi function sites.	Care Homes (codes included: joint user hospital, contractual hospital, home for the elderly, other home, miscellaneous premises, care home, private nursing home, private hospital)**	Care home
Hospices include Sue Ryder Homes; Marie Curie Centres; oncology centres; voluntary hospice units; and palliative care centres.		Hospice
Other Communal Establishments include schools for people with learning disabilities; holiday homes and hotels; common lodging houses; aged persons' accommodation; assessment centres; schools; convents and monasteries; nurses' homes; university and college halls of residence; young offender institutions; secure training centres; detention centres; prisons and remand homes.	Other institutions include clinics, medical centres, prisons and schools. (codes included: GP surgery, health centre, medical centre, clinic premises, primary school, non-NHS maternity, prisons, school)***	Other includes deaths at a residential address which was not the usual address of the deceased
Elsewhere includes all places not covered above such as deaths on a motorway; at the beach; climbing a mountain; walking down the street; at the cinema; at a football match; while out shopping; or in someone else's home. This category also includes people who are pronounced dead on arrival at hospital.		

*May include hospices located on hospital sites where no separate location code exists

**May include hospices located separate from hospital site where the hospice has a unique location code

***Unclear whether this category will include some hospice sites

Appendix Figure 1. All deaths registered in the UK during the COVID-19 pandemic between week 11 of 2020 and week 10 of 2021 (07/03/2021 to 12/03/2021 in England, Wales and Northern Ireland, and 09/03/2021 to 14/03/2021 in Scotland) death rate per 100,000 population by nation

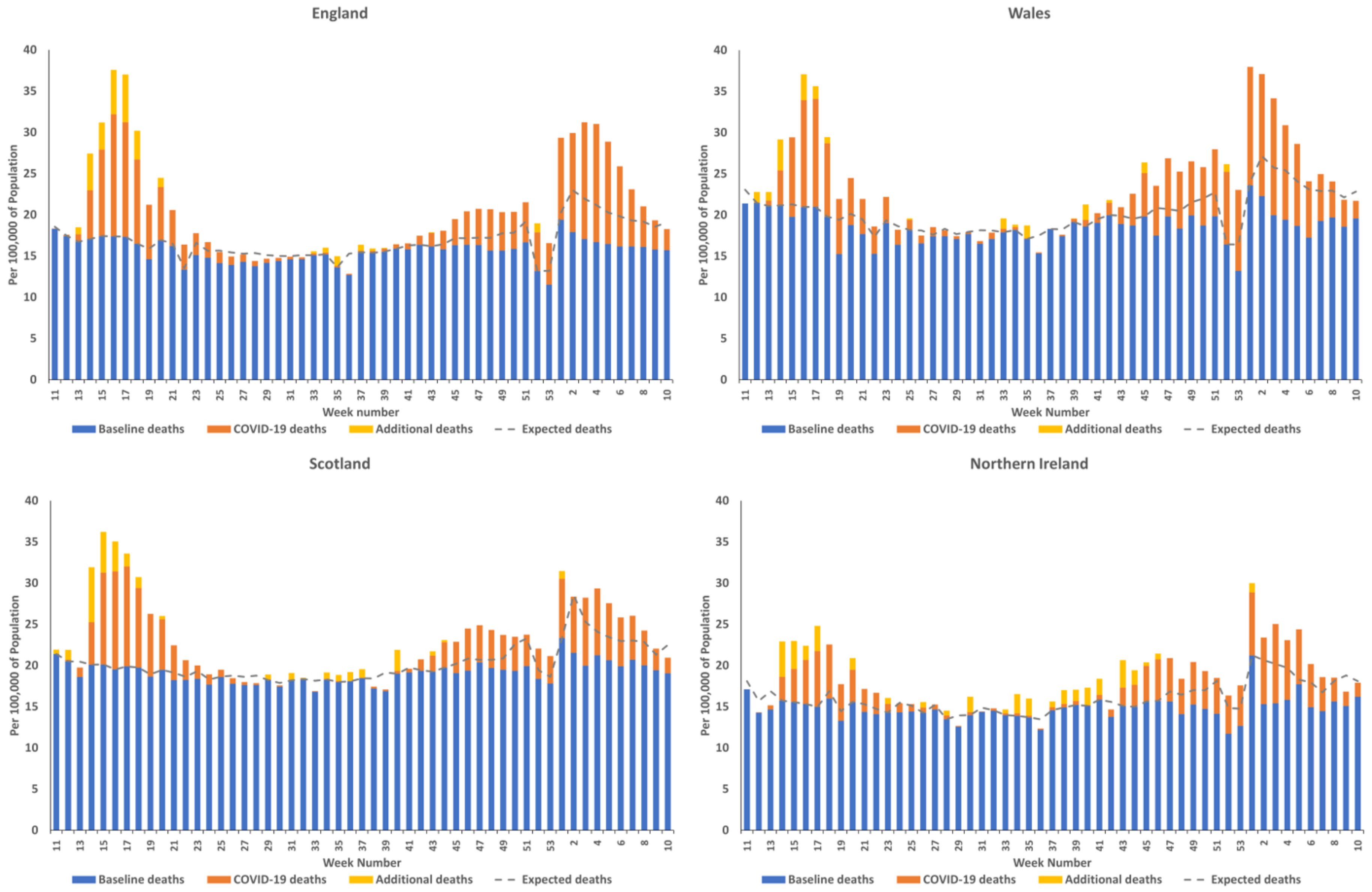

Appendix Figure 2. Deaths registered in England during the COVID-19 pandemic between week 11 of 2020 and week 10 of 2021 (07/03/2021 to 12/03/2021) death rate per 100,000 population by region

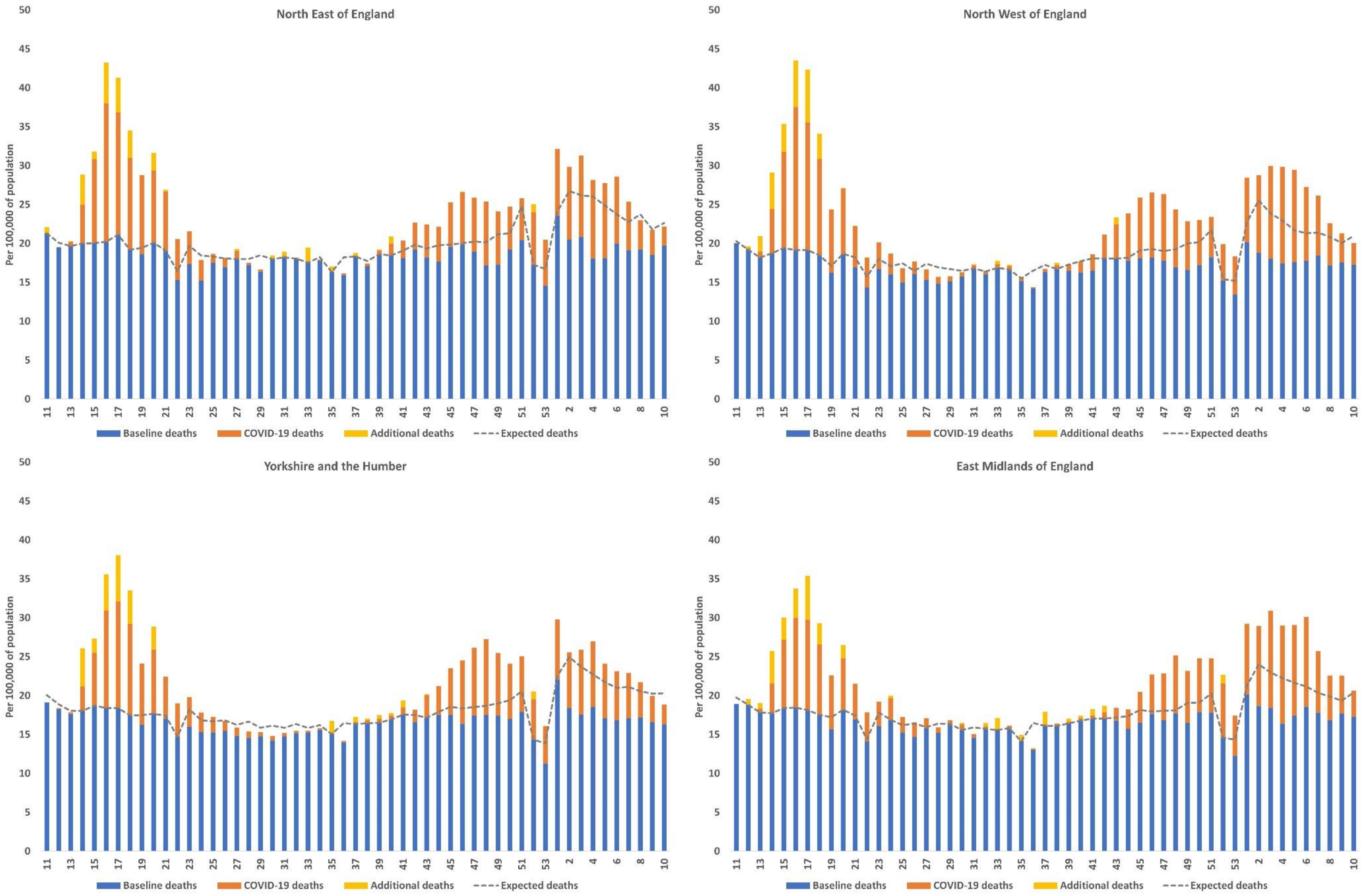

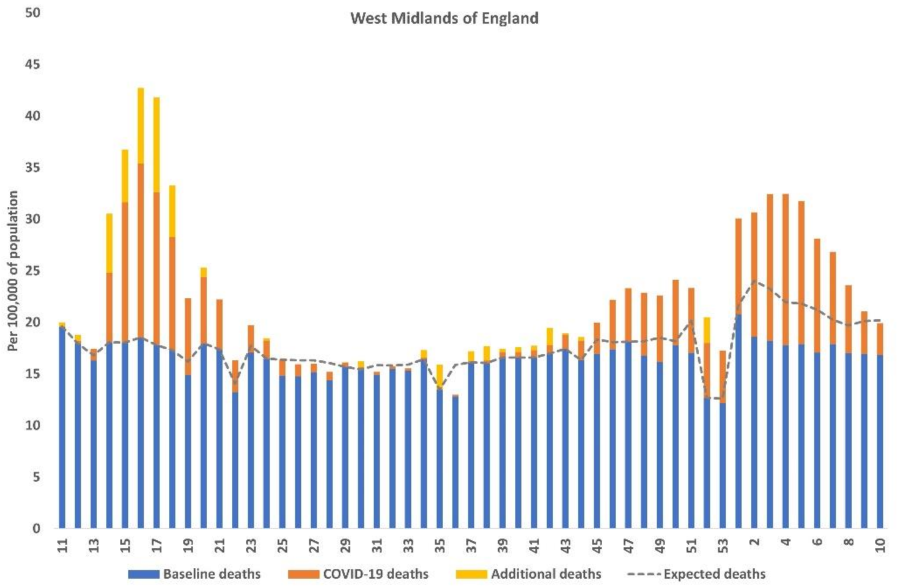
West Midlands of England
Per 100,000 of population
0 5 10 15 20 25 30 35 40 45 50
11 13 15 17 19 21 23 25 27 29 31 33 35 37 39 41 43 45 47 49 51 53 2 4 6 8 10
Baseline deaths
COVID-19 deaths
Additional deaths
Expected deaths

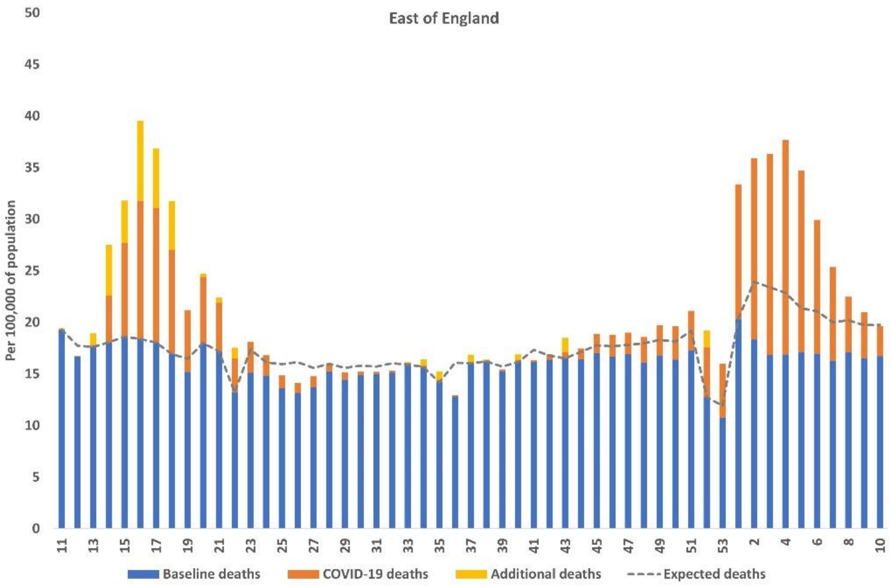
East of England
Per 100,000 of population
0 5 10 15 20 25 30 35 40 45 50
11 13 15 17 19 21 23 25 27 29 31 33 35 37 39 41 43 45 47 49 51 53 2 4 6 8 10
Baseline deaths
COVID-19 deaths
Additional deaths
Expected deaths

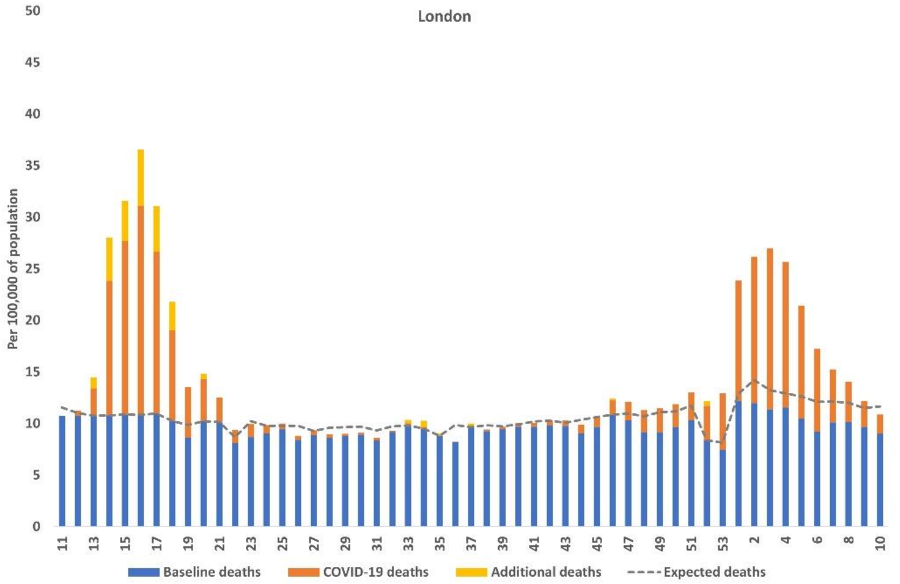
London
Per 100,000 of population
0 5 10 15 20 25 30 35 40 45 50
11 13 15 17 19 21 23 25 27 29 31 33 35 37 39 41 43 45 47 49 51 53 2 4 6 8 10
Baseline deaths
COVID-19 deaths
Additional deaths
Expected deaths

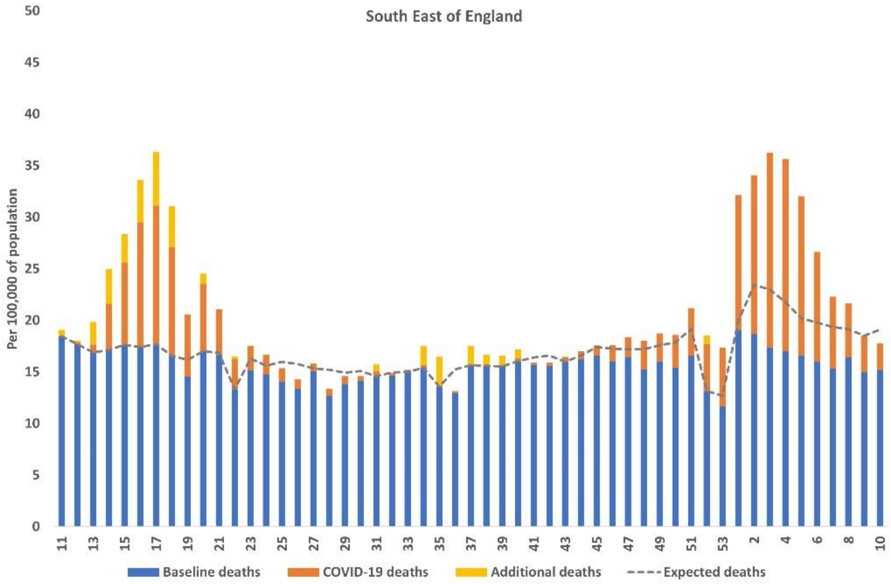
South East of England
Per 100,000 of population
0 5 10 15 20 25 30 35 40 45 50
11 13 15 17 19 21 23 25 27 29 31 33 35 37 39 41 43 45 47 49 51 53 2 4 6 8 10
Baseline deaths
COVID-19 deaths
Additional deaths
Expected deaths

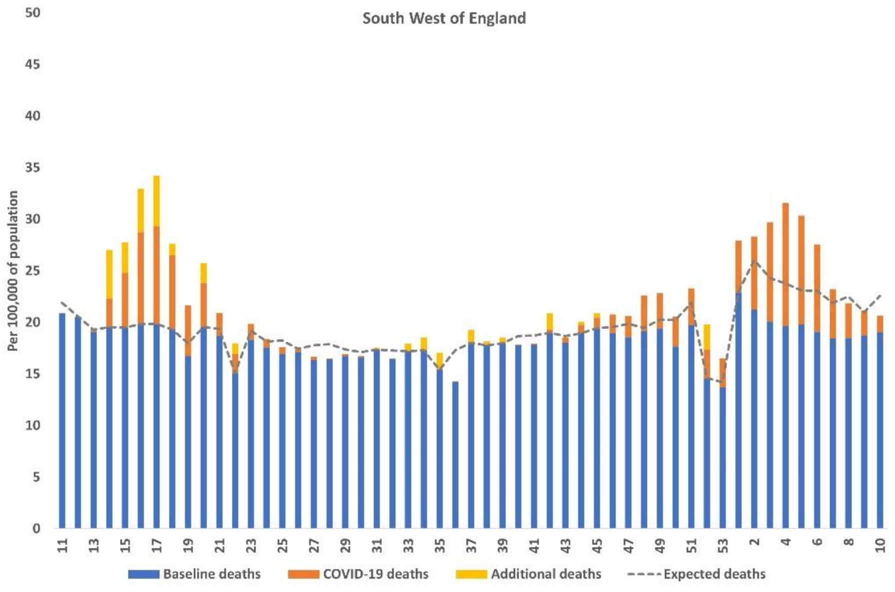
South West of England
Per 100,000 of population
50
45
40
35
30
25
20
15
10
5
0
11
13
15
17
19
21
23
25
27
29
31
33
35
37
39
41
43
45
47
49
51
53
2
4
6
8
10
Baseline deaths
COVID-19 deaths
Additional deaths
Expected deaths

Appendix Text 2. Summary of mortality patterns analysis of the regions of England

Within England, there was a disparity in COVID-19 deaths between regions, particularly comparing the regions of Southern and Northern England (Appendix Table 2 and Appendix Figure 1). Over the study period, the death rate attributed to COVID-19 was greatest in the North West at 269 per 100,000, and lowest in the South West at 142 per 100,000. The additional death rate not attributed to COVID-19 ranged from 24 per 100,000 in London to 46 per 100,000 in the West Midlands. In London over the study period, the total death rate was 748 per 100,000, the lowest in England. However, the average death rate during the same timeframe in 2015-19 was lower in London at 560 per 100,000 compared to a range in the rest of England of between 898 and 1066 per 100,000. Therefore, the percentage increase in total deaths over the study interval is higher in London at 34% compared the rest of England (ranging between 11% in the South West to 25% in the West Midlands).

Appendix Table 2. Death rate per 100,000 population during the COVID-19 pandemic between week 11 of 2020 and week 10 of 2021 (07/03/2020 to 12/03/2021) in the regions of England, Wales, Northern Ireland and (09/02/2020 to 14/03/2021) in Scotland

Nation of the UK/Region of England	Average death rate* (2015-19) per 100,000	Total death rate* per 100,000	COVID-19 death rate* per 100,000	Additional death rate* per 100,000	Percentage increase in total deaths*
**England**	895	1086	225	29	21%
**North East**	1066	1263	253	27	18%
**North West**	990	1205	269	28	22%
**Yorkshire and The Humber**	962	1137	225	30	18%
**East Midlands**	947	1148	233	30	21%
**West Midlands**	935	1172	250	46	25%
**East**	928	1127	237	36	21%
**London**	560	748	209	24	34%
**South East**	898	1086	221	34	21%
**South West**	1025	1140	142	31	11%
**Wales**	1073	1251	244	19	17%
**Scotland**	1073	1223	180	30	12%
**Northern Ireland**	847	976	152	38	15%

*Over the study period

Appendix Table 3. Age distribution of average deaths (2015-2019), COVID-19 deaths and additional deaths in the UK (England, Wales, Scotland and Northern Ireland) during the COVID-19 pandemic between week 11 of 2020 and week 6 of 2021 (07/03/2021 to 12/02/2021 in England, Wales and Northern Ireland, and 09/03/2021 to 14/02/2021 in Scotland) split according to nation

Mortality Category	Age Category	England and Wales	Scotland	Northern Ireland	United Kingdom
**Average Deaths (2015-2019)**	**<45**	3%	4%	5%	4%
	**45-64**	12%	14%	14%	12%
	**65-74**	17%	18%	17%	17%
	**75-84**	28%	30%	29%	28%
	**85+**	40%	33%	36%	39%
**COVID-19 Deaths**	**<45**	1%	1%	1%	1%
	**45-64**	10%	10%	8%	10%
	**65-74**	15%	16%	14%	15%
	**75-84**	32%	33%	34%	32%
	**85+**	42%	41%	43%	42%
**Additional Deaths**	**<45**	2%	11%	9%	2%
	**45-64**	15%	23%	21%	16%
	**65-74**	10%	18%	17%	10%
	**75-84**	27%	22%	26%	26%
	**85+**	46%	27%	27%	45%

Appendix Table 4. Age distribution of average deaths (2015-2019), COVID-19 deaths and additional deaths in the UK (England, Wales, Scotland and Northern Ireland) during the COVID-19 pandemic between week 11 of 2020 and week 6 of 2021 (07/03/2021 to 12/02/2021 in England, Wales and Northern Ireland, and 09/03/2021 to 14/02/2021 in Scotland) split according to waves of the pandemic

Mortality Category	Age Category	Wave One[a]	Trough[b]	Wave Two[c]	Total[*]
**Average Deaths (2015-2019)**	**<45**	4%	4%	3%	4%
	**45-64**	12%	13%	12%	12%
	**65-74**	17%	17%	16%	17%
	**75-84**	28%	29%	28%	28%
	**85+**	39%	38%	40%	39%
**COVID-19 Deaths**	**<45**	1%	1%	1%	1%
	**45-64**	10%	9%	10%	10%
	**65-74**	15%	15%	16%	15%
	**75-84**	32%	33%	31%	32%
	**85+**	42%	42%	42%	42%
**Additional Deaths**	**<45**	0.1%	6%	15%	2%
	**45-64**	8%	36%	61%	16%
	**65-74**	9%	13%	18%	10%
	**75-84**	27%	33%	4%	26%
	**85+**	56%	11%	1%	45%

a. Wave one = week 14 to 22 of 2020 (28/03/2020 to 29/05/2020 in England and Wales, 30/03/2020 to 31/05/2020 in Scotland) – 9 weeks total length

b. Trough = week 23 to 42 of 2020 (30/05/2020 to 16/10/2020 in England and Wales, 01/06/2020 to 18/10/2020 in Scotland) – 20 weeks total length

c. Wave two = week 43 of 2020 to week 6 of 2021 (17/10/2020 to 12/02/2021 in England and Wales, 19/10/20 to 14/02/2021 in Scotland) – 17 weeks total length

*During the whole study period between week 11 of 2020 and week 6 of 2021

Appendix Figure 3. COVID-19 and non-COVID deaths registered as occurring in hospitals in the UK (England, Wales, Scotland and Northern Ireland) during the COVID-19 pandemic between week 11 of 2020 and week 10 of 2021 (07/03/2021 to 12/03/2021 in England, Wales and Northern Ireland, and 09/03/2021 to 14/03/2021 in Scotland), according to nation

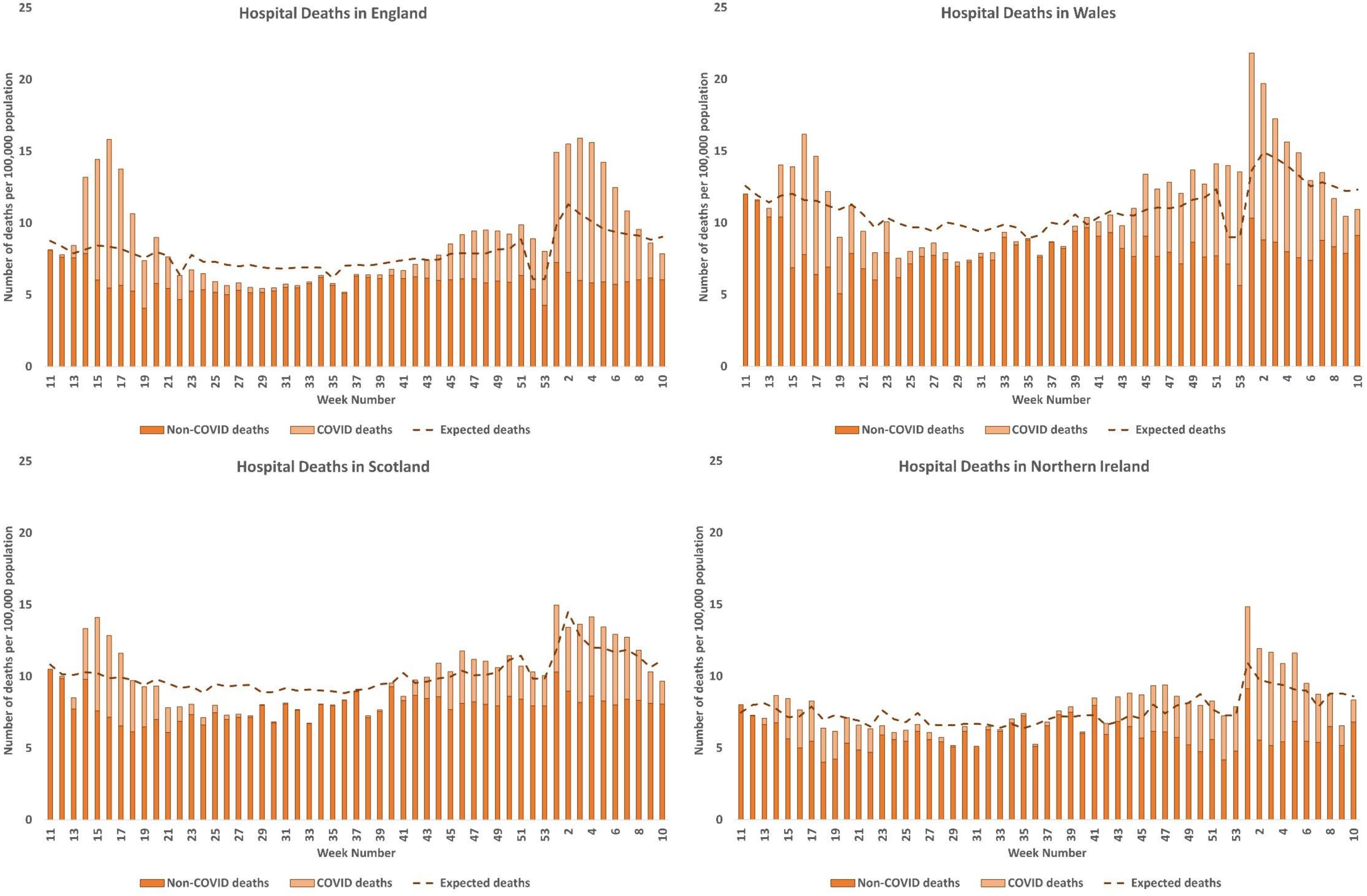

Appendix Figure 4. COVID-19 and non-COVID deaths registered as occurring in homes in the UK (England, Wales, Scotland and Northern Ireland during the COVID-19 pandemic between week 11 of 2020 and week 10 of 2021 (07/03/2021 to 12/03/2021 in England, Wales and Northern Ireland, and 09/03/2021 to 14/03/2021 in Scotland), according to nation

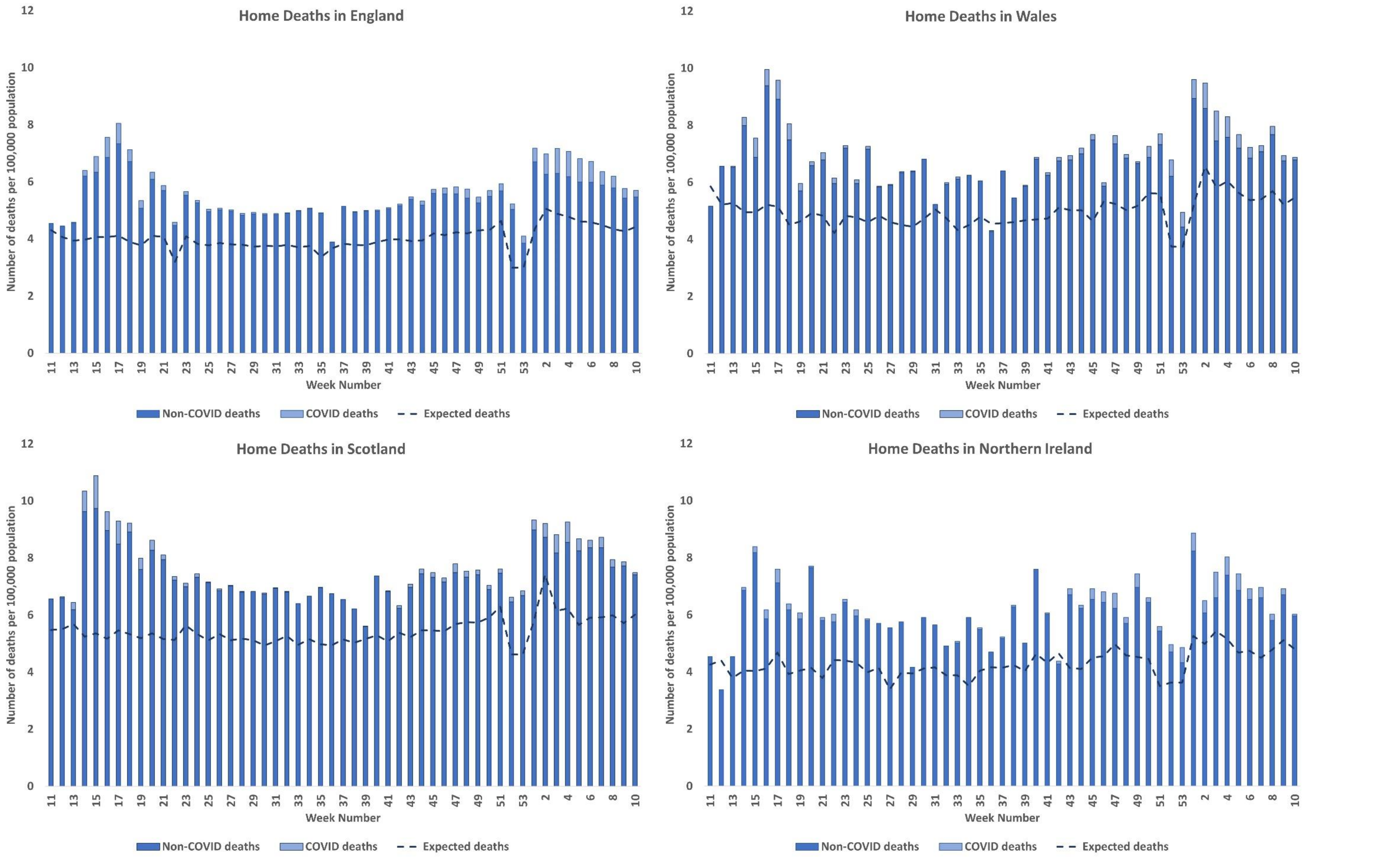

Appendix Figure 5. COVID-19 and non-COVID deaths registered as occurring in care homes in the UK (England, Wales, Scotland and Northern Ireland during the COVID-19 pandemic between week 11 of 2020 and week 10 of 2021 (07/03/2021 to 12/03/2021 in England, Wales and Northern Ireland, and 09/03/2021 to 14/03/2021 in Scotland), according to nation

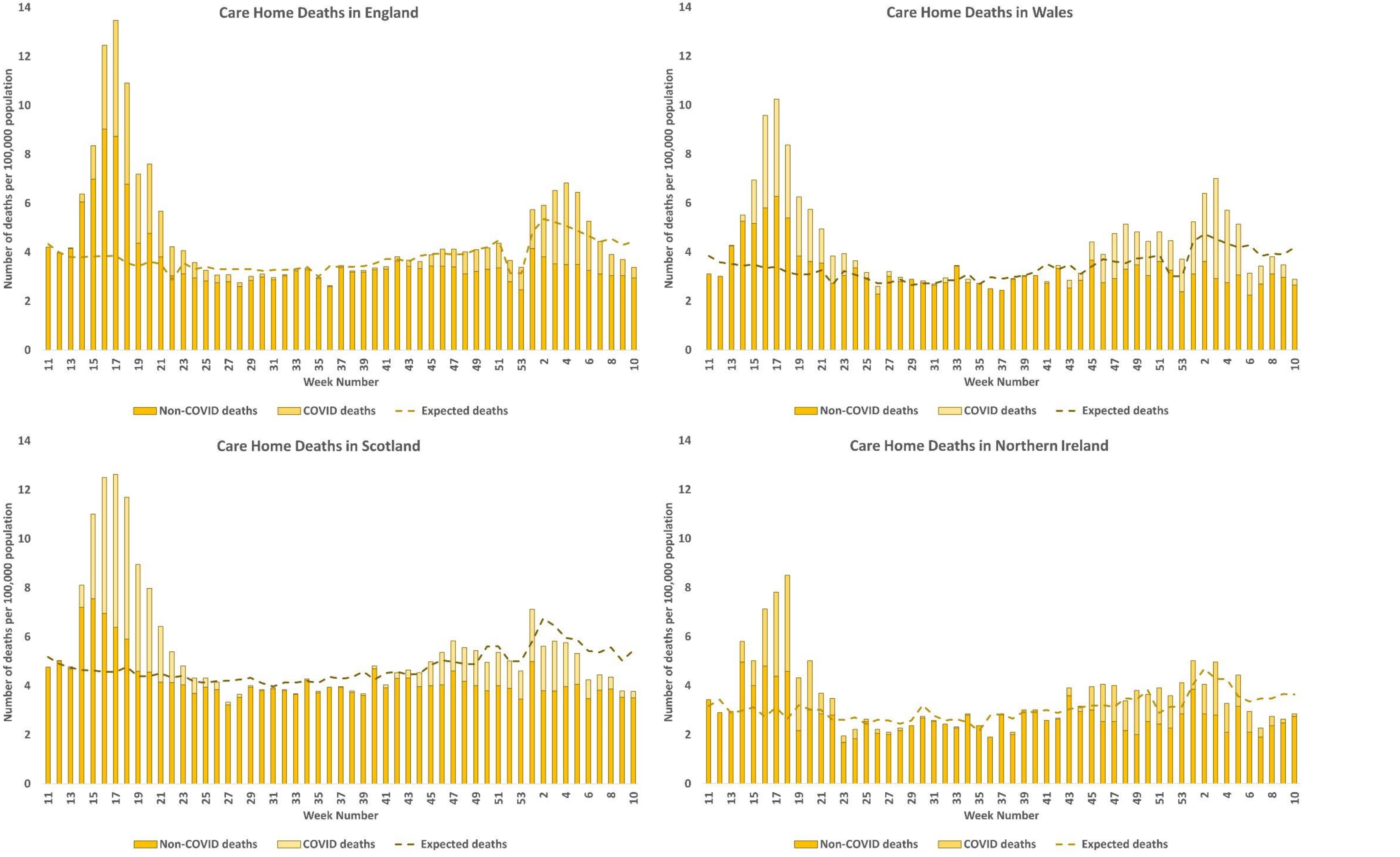

Appendix Figure 6. COVID-19 and non-COVID deaths registered as occurring in hospices in England, Wales and Northern Ireland during the COVID-19 pandemic between week 11 of 2020 and week 10 of 2021 (07/03/2021 to 12/03/2021 in England, Wales and Northern Ireland, and 09/03/2021 to 14/03/2021 in Scotland), according to nation

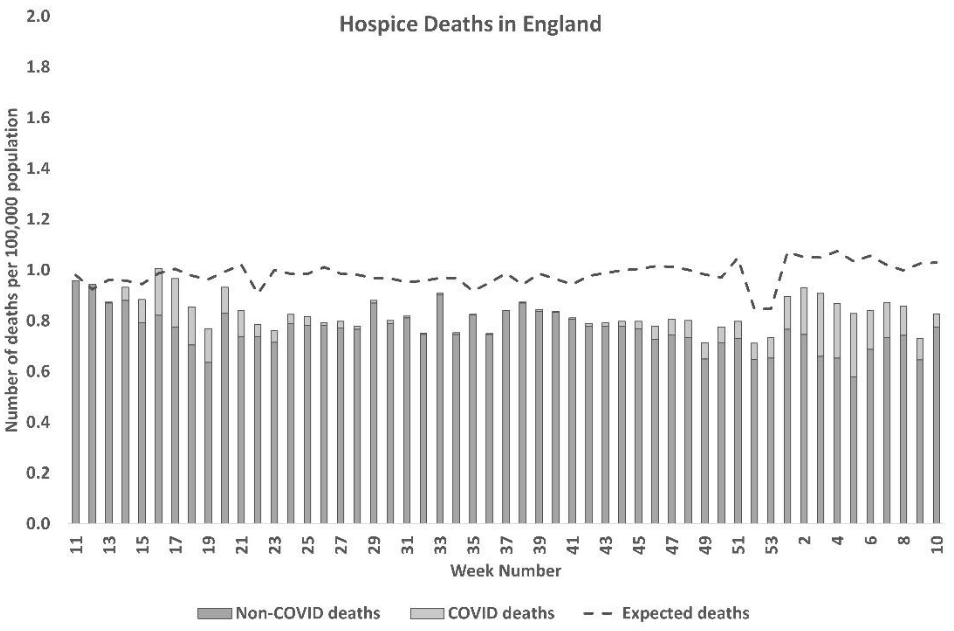

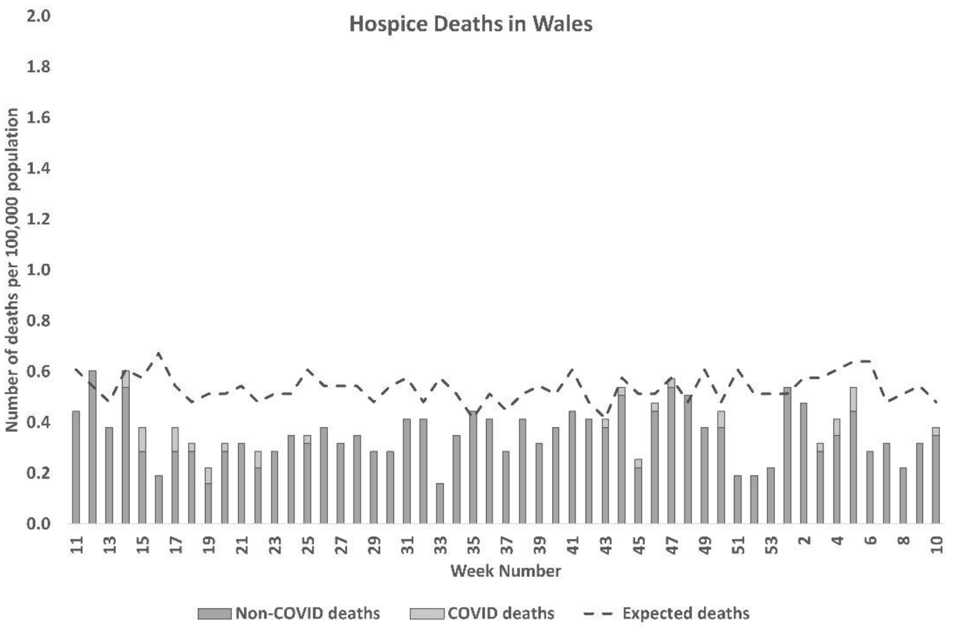

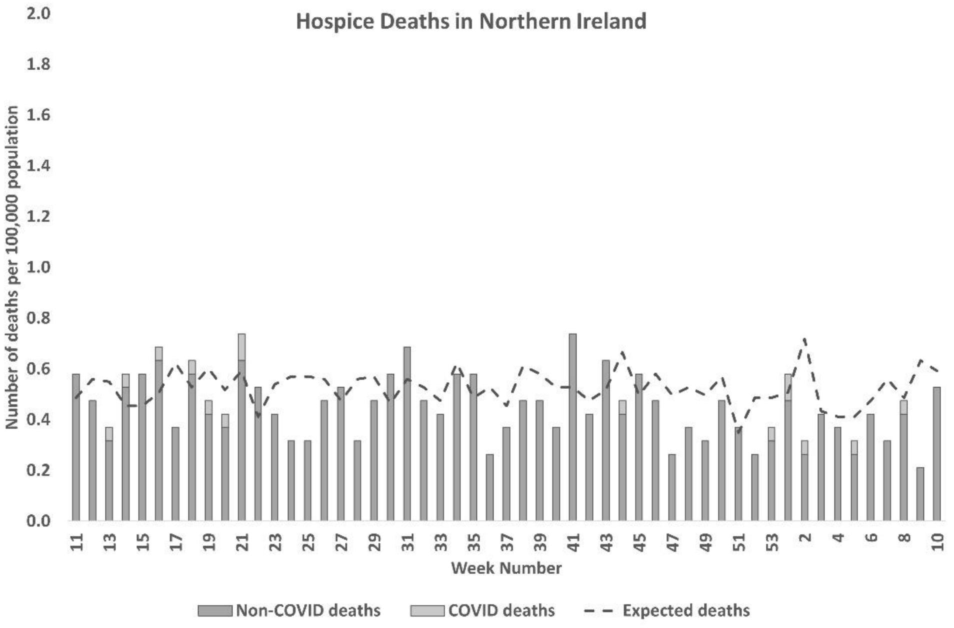

Appendix Figure 7. All deaths registered in the UK during the COVID-19 pandemic between week 11 of 2020 and week 10 of 2021 (07/03/2021 to 12/03/2021 in England, Wales and Northern Ireland, and 09/03/2021 to 14/03/2021 in Scotland) according to place of death by relative proportion

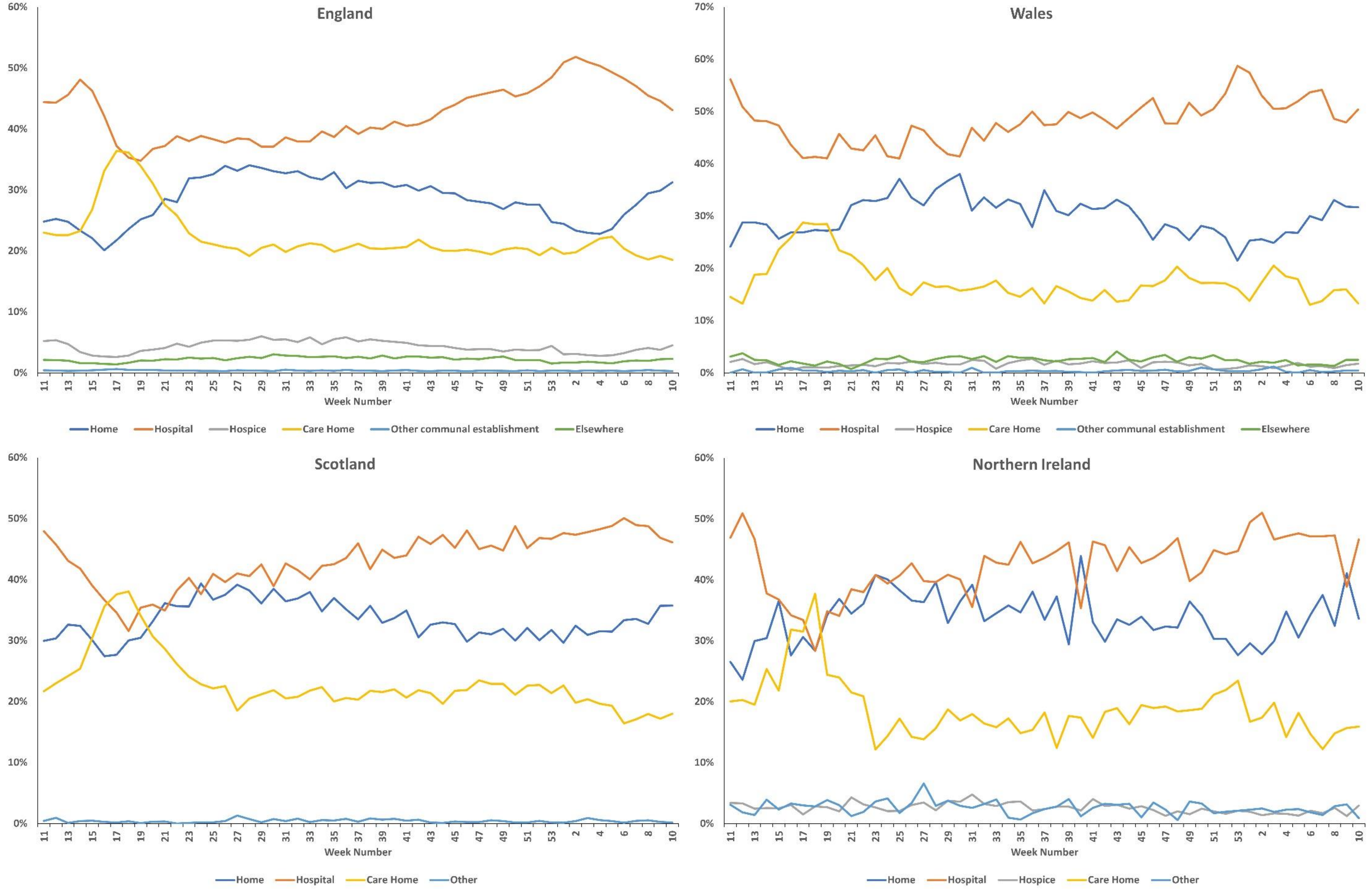

Appendix Text 3. Summary of place of death analysis by individual UK nation

Overall, most deaths occurred in hospital during the pandemic (Appendix Figure3). The percentage of deaths occurring in hospital over the study period was 43% in England, 48% in Wales, 43% in Scotland and 42% in Northern Ireland. Deaths at home accounted for a significant proportion of deaths registered during the study period (see Appendix Figure 4). Of all deaths registered, the percentage occurring at home was 27% in England, 29% in Wales, 33% in Scotland, and 33% in Northern Ireland. Care homes were the next most common place for people to die (Appendix Figure 5). Of all deaths registered, deaths in care homes accounted for 23% of deaths in England, 18% in Wales, 23% in Scotland, and 19% in Northern Ireland. Over the study period, deaths in hospices accounted for 4% of all deaths in England, 2% in Wales, and 2% in Northern Ireland (see Appendix Figure 6).

Most COVID-19 deaths occurred in hospital. Of all COVID-19 deaths registered, the percentage of deaths in hospital during the study period was 69% in England, 72% in Wales, 60% in Scotland, and 66% in Northern Ireland. Many COVID-19 deaths occurred in care homes, the percentage of COVID-19 deaths occurring in care homes during the study period was 23% in England, 21% in Wales, 33% in Scotland and 27% in Northern Ireland. Few COVID-19 deaths took place in hospices, the percentage of COVID-19 deaths in hospices during the study period was 2% in England, 0.4% in Wales, and 1% in Northern Ireland. Separate data for deaths in hospices in Scotland was not available for analysis.

Compared to the average of the previous five years, the number of deaths at home increased by 40% in Wales, 41% in England and Scotland, and 43% in Northern Ireland. Hospital deaths increased by 3% in Northern Ireland, 4% in Wales, and 13% in England but decreased by 1% in Scotland. Fewer people died in hospices throughout the study period amounting to a fall of 13% in Northern Ireland, 14% in England and 31% in Wales. Care home deaths increased by 14% in Scotland and Northern Ireland, 25% in England and 26% in Wales during the study period (Appendix Table 6).

There were also variations in the relative proportions of place of death throughout the pandemic (Appendix Figure 7). In week 17 in Scotland and week 18 in England and Northern Ireland, care homes became the most common place to die, briefly overtaking hospitals, while this never occurred in Wales. In Scotland, the proportion of deaths taking place at home exceeded hospital deaths in weeks 21 and 24. This also occurred in Northern Ireland in weeks 20, 24, 31, and 40, though due to the lower numbers of deaths in Northern Ireland compared to the rest of the UK, there is greater variance in the data.

Appendix Figure 7. Daily testing capacity at week end from week 15 of 2020 to week 6 of 2021 (10/04/2020 to 12/02/2021) and actual tests conducted on the final day of each week between week 17 of 2020 to week 6 of 2021 (24/04/2021 to 12/02/2021)[4]

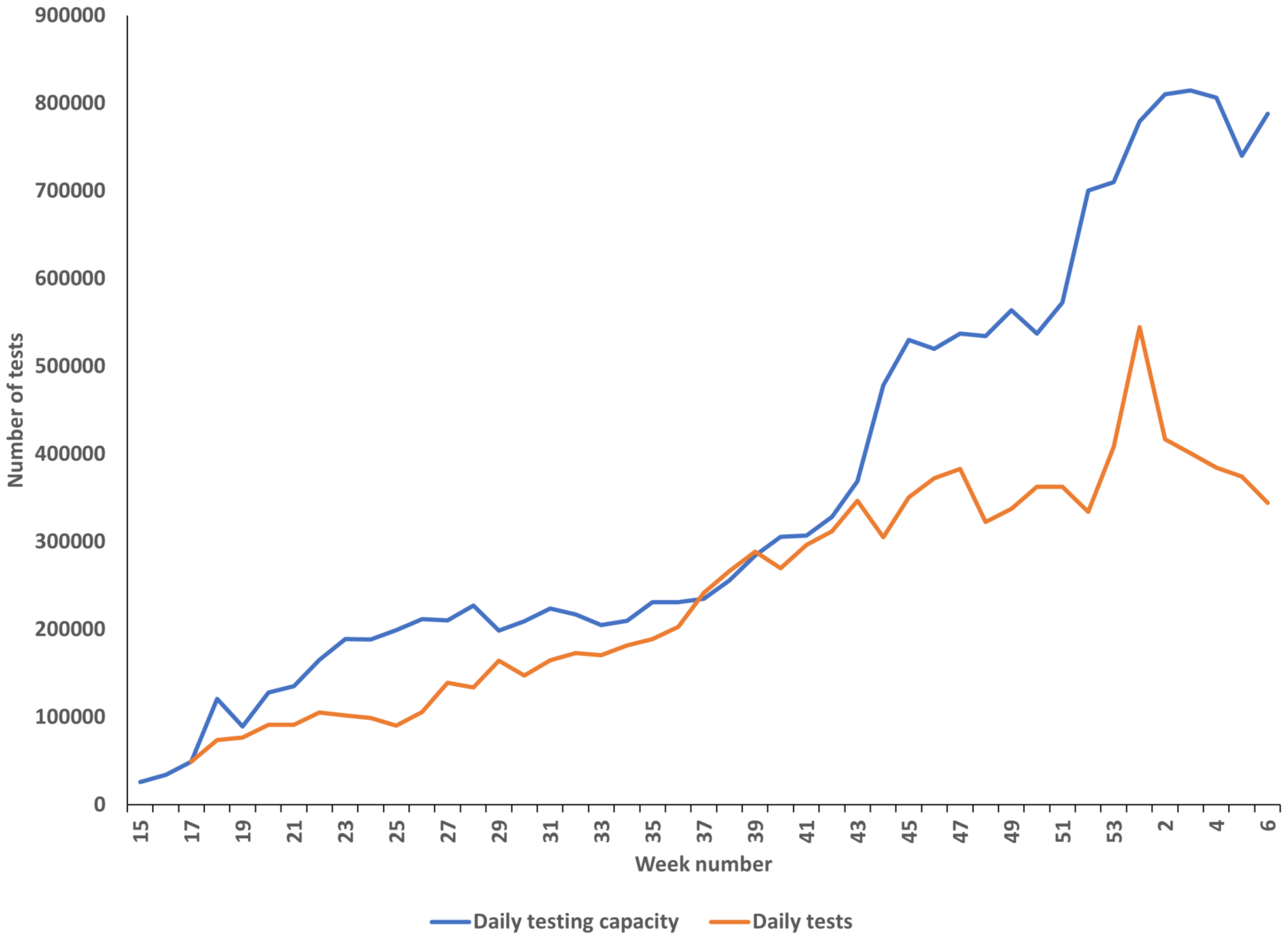

Appendix Table 5. Care home demographics and percentage of population aged 75 years and older for the four UK nations

Nation	Percentage of population ≥75	Care home places	Places per 100,000 population	Places per 100 people aged ≥75 years
England	8[1]	457,295[5]	812	9.6
Wales	10[1]	25,493[6]	809	8.5
Scotland	9[2]	41,032[7]	751	8.8
Northern Ireland	8[3]	16,080[8]	849	11.2

Appendix Table 6. Percentage change in place of death during the COVID-19 pandemic between week 11 of 2020 and week 10 of 2021 (07/03/2020 to 12/03/2021) in England, Wales, Northern Ireland and (09/02/2020 to 14/03/2021) in Scotland compared to the 5-year average (2015-2019)

**Place of death**	**Nation**			
	**England**	**Wales**	**Scotland**	**Northern Ireland**
**Home**	+41%	+40%	+41%	+43%
**Hospital**	+13%	+4%	-1%	+3%
**Care Home**	+25%	+26%	+14%	+14%
**Hospice**	-14%	-31%		-13%

References

1. Office for National Statistics. Deaths registered weekly in England and Wales, provisional, https://www.ons.gov.uk/peoplepopulationandcommunity/birthsdeathsandmarriages/deaths/datasets/weeklyprovisionalfiguresondeathsregisteredinenglandandwales (2021, accessed 01/04/2021).
2. National Records of Scotland. Deaths involving coronavirus (COVID-19) in Scotland, https://www.nrscotland.gov.uk/covid19stats (2021, accessed 01/04/2021).
3. Northern Ireland Statistics and Research Agency. Weekly death registrations in Northern Ireland, 2020, https://www.nisra.gov.uk/publications/weekly-deaths (2021, accessed 01/04/2020).
4. Public Health England. Coronavirus in the UK: Testing in United Kingdom, https://coronavirus.data.gov.uk/details/testing (2021, accessed 30/03/2021).
5. Public Health England. Palliative and End of Life Care Profiles: Care Homes and Community, https://fingertips.phe.org.uk/profile/end-of-life/data#page/11/gid/1938133060/pat/159/par/E92000001/ati/15/are/E92000001/iid/92489/age/162/sex/4/cid/4/tbm/1/page-options/ovw-do-0_eng-vo-0_eng-do-0 (2020, accessed 01/03/2021).
6. Statistics for Wales. CIW Services and Places by Setting Type and Year, https://statswales.gov.wales/Catalogue/Health-and-Social-Care/Services-for-Social-Care-and-Childrens-Day-Care/cssiwservicesandplaces-by-setting-year (2021, accessed 01/03/2021).
7. Public Health Scotland. *Care Home Census for Adults in Scotland*. 27/10/2020.
8. Health and Social Care Northern Ireland. *Northern Ireland COVID-19 Regional Action Plan for the Care Home Sector*. 06/09/2020.
